# Functional Compression Fabrics with Dual Scar-Suppressing and Antimicrobial Properties: Microencapsulation Design and Performance Evaluation

**DOI:** 10.3390/jfb16080287

**Published:** 2025-08-05

**Authors:** Lihuan Zhao, Changjing Li, Mingzhu Yuan, Rong Zhang, Xinrui Liu, Xiuwen Nie, Bowen Yan

**Affiliations:** 1 School of Textile Science and Engineering, Tiangong University, Tianjin 300387, China; lcjing5629@163.com (C.L.); ymz0012@126.com (M.Y.); zr07032024@163.com (R.Z.); 17796982832@163.com (X.L.); 15502632618@163.com (X.N.); yanbowen06@163.com (B.Y.); 2 Ministry of Education Key Laboratory for Advanced Textile Composite Materials, Tiangong University, Tianjin 300387, China

**Keywords:** compression garment fabric, polydimethylsiloxane, chitosan quaternary ammonium salt, sodium alginate, microcapsule, antibacterial performance

## Abstract

Pressure therapy combined with silicone has a significant effect on scar hyperplasia, but limitations such as long-term wearing of compression garments (CGs) can easily cause bacterial infection, cleanliness, and lifespan problems of CGs caused by the tedious operation of applying silicone. In this study, a compression garment fabric (CGF) with both inhibition of scar hyperplasia and antibacterial function was prepared. A polydimethylsiloxane (PDMS)-loaded microcapsule (PDMS-M) was prepared with chitosan quaternary ammonium salt (HACC) and sodium alginate (SA) as wall materials and PDMS as core materials by the complex coagulation method. The PDMS-Ms were finished on CGF and modified with (3-aminopropyl)triethoxysilane (APTES) to obtain PDMS-M CGF, which was further treated with HACC to produce PDMS-M-HACC CGF. X-ray Photoelectron Spectroscopy(XPS) and Fourier transform infrared spectroscopy (FTIR) analysis confirmed the formation of covalent bonding between PDMS-M and CGF. The PDMS-M CGF exhibited antibacterial rates of 94.2% against Gram-negative bacteria *Escherichia coli* (*E. coli*, AATCC 6538) and of 83.1% against Gram-positive bacteria *Staphylococcus aureus* (*S. aureus*, AATCC 25922). The antibacterial rate of PDMS-M-HACC CGF against both *E. coli* and *S. aureus* reached 99.9%, with wash durability reaching grade AA for *E. coli* and approaching grade A for *S. aureus*. The finished CGF maintained good biocompatibility and showed minimal reduction in moisture permeability compared to unfinished CGF, though with decreased elastic recovery, air permeability and softness. The finished CGF of this study is expected to improve the therapeutic effect of hypertrophic scars and improve the quality of life of patients with hypertrophic scars.

## 1. Introduction

Burns, trauma, and other wounds are prone to developing hypertrophic scars during the healing process [1]. The main clinical manifestations include raised, thickened, and hardened scar tissue that appears as irregular red protrusions, often accompanied by burning sensations, pain, pruritus, and other uncomfortable symptoms [2,3], which significantly impair patients’ quality of life, physical condition, and psychological well-being [4].

Current clinical treatments for hypertrophic scars include pressure therapy [5,6,7], laser therapy [8,9,10], silicone therapy [11,12], surgical treatment [13,14], and pharmacotherapy [15,16,17]. Studies have demonstrated that combination therapy using multiple modalities is significantly more effective than monotherapy [18,19]. Among these, the combination of silicone (polydimethylsiloxane, PDMS) and pressure therapy is widely used in clinical practice [20]. Silicone therapy [21] involves covering the scar surface with silicone-based gels or dressings, which exert anti-fibrotic effects, soften scars, and reduce hyperpigmentation. Pressure therapy [22] applies continuous pressure to healing scar tissue via compression garments, inducing localized hypoxia in the scar tissue and slowing collagen production, thereby reducing scar proliferation. The combination of these two therapies compensates for silicone’s limited inhibitory effect on fibroblast growth while improving overall treatment efficacy. It also addresses the issue of pressure therapy causing friction and potential wound damage to dry skin. A controlled trial involving 104 patients with incision scar hyperplasia showed that silicone gel dressing combined with pressure therapy led to significantly better outcomes compared to pressure therapy alone at 2- and 6-month follow-ups, with the Vancouver Scar Assessment Scale showing the most significant improvements in scar color and vascularity [20]. Chow et al. [23] developed three-dimensional custom-fabricated silicone composites for pressure therapy that deliver targeted pressure to scar sites while maintaining comfort in unaffected areas. However, despite its proven efficacy, the current combination of pressure and silicone therapy presents several challenges, including bacterial growth and infection risks from prolonged compression garment (CG) use [24], and the operation of applying silicone, which is cumbersome and affects the cleanliness and lifespan of the CG.

To address these issues, researchers have explored antimicrobial modifications for CG. Zhao et al. [2] developed graphene oxide/chitosan antimicrobial compression fabric (CGF) and achieved antibacterial rates of 92.09% and 99.33% against *E. coli* and *S. aureus*, respectively. Similarly, Li et al. [24] prepared an antimicrobial compression garment fabric based on Ti3C2Tx and (3-aminopropyl)triethoxysilane (APTES). The results showed that the unwashed antibacterial compression garment fabric exhibited an antibacterial rate of 99.9% against *S. aureus* and of 99.76% against *E. coli*. The wash fastness of the antibacterial fabric met the industry-standard grade AA, and the abrasion resistance of the treated fabric was sufficient for daily wear requirements.

To combine silicone and antibacterial CGFs, thereby realizing silicone therapy combined with pressure therapy while preventing bacterial infection in affected areas and simplifying silicone application procedures, silicone can be microencapsulated as the core material and incorporated into CGFs, with its efficacy maintained through sustained release. Microencapsulation technology involves encapsulating highly active, volatile, or functional solids/liquids within stable membrane materials to form microparticles [25,26]. This technology has been widely applied in cosmetics and drug delivery systems due to its ability to control the release of encapsulated substances in response to various stimuli, including friction, pressure, and temperature [27]. Chitosan quaternary ammonium salt (HACC), a chitosan derivative, exhibits superior water solubility and antimicrobial activity compared to native chitosan [28]. Sodium alginate (SA) is a natural, non-toxic, anionic polymer possessing excellent biodegradability, biocompatibility, and tissue-adhesive properties [29]. The electrostatic interaction between HACC’s quaternary ammonium groups and SA’s carboxyl groups facilitates effective microcapsule formation [28,30]. Topical silicone [31] can form a hydration film on the surface of scars, regulate the hydration of scar tissue, reduce collagen deposition, and improve scar color, flatness and texture, and it is widely used in the early prevention and treatment of various scars.

Therefore, HACC and SA can be used as wall material and PDMS as the core material to prepare microcapsules for finishing the CGF. This CG with a PDMS microcapsule finish can not only exert pressure on the affected area but also improve scar pliability and pigmentation. Additionally, HACC as the wall material provides antimicrobial effects, which can avoid the risk of bacterial growth and wound infection.

However, clinical practice and further studies have shown that the risk of infection in burn patients is extremely complex and severe [32,33,34]. Not only is post-burn skin an ideal breeding ground for bacteria, but there is also much uncertainty about the source and extent of infection over time and during treatment. Although microencapsulated finished CGF already has a certain degree of antimicrobial capacity, its antimicrobial properties need to be further strengthened to address possible drug-resistant bacteria and high-intensity infection risks. HACC, as a cationic macromolecule polymer, possesses broad-spectrum antimicrobial activity and has a unique antimicrobial mechanism capable of destroying bacterial cell membrane integrity and interfering with bacterial metabolic processes, thus efficiently inhibiting bacterial growth. By further finishing HACC on microencapsulated CGF, it is expected to build a more powerful and multilayered antimicrobial defense. This can not only enhance the immediate killing ability of CGs against bacteria but also synergize with the microencapsulation system to achieve comprehensive antibacterial protection through different time scales and modes of action.

**Figure 1:**
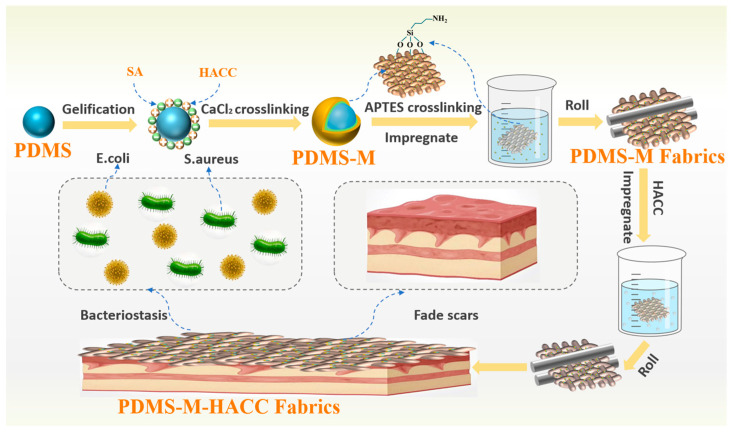
The preparation process of PDMS-M-HACC CGF.

To endow CGF with both scar-inhibition and antibacterial functionalities, this study developed a novel multifunctional CGF. First, PDMS-Ms were prepared by the compound coalescence method using SA and HACC as the wall material and PDMS as the core material. Then, the prepared PDMS-Ms were prepared on CGF by the dipping–rolling method, and the coated fabric was modified using (3-aminopropyl)triethoxysilane (APTES) to enhance the connection between the microcapsules and the CGF (the silane groups generated by APTES hydrolysis can bind with the wall material of the microcapsules, HACC, SA, and hydroxyl groups on the CGF surface), resulting in PDMS-M CGF. Furthermore, to enhance the antibacterial performance of the PDMS-M CGF, HACC was re-dipped on the PDMS-M CGF to obtain PDMS-M-HACC CGF (the preparation process is illustrated in Figure 1). Finally, the antibacterial performance, washing fastness, wearing performance, and biocompatibility of both PDMS-M CGF and PDMS-M-HACC CGF were investigated. The functional CGF prepared in this study can not only sustain the release of PDMS through microcapsules, achieving the effect of inhibiting scar hyperplasia and fading scars, but also provide excellent antibacterial effects through HACC. The results of this study will provide a more effective solution for the treatment of hypertrophic scars and provide protection for improving the quality of life of patients. This study provides novel technical approaches and theoretical foundations for the development of functional medical textiles.

## 2. Experimental Section

### 2.1. Materials

HACC (98% substitution degree), SA (AR,M/G = 1:2), glacial acetic acid (HAC) (AR), and sodium hydroxide (NaOH) (AR) were purchased from Tianjin One Way Science and Technology Co., Ltd. (Tianjin, China). Tween-80 (AR) was obtained from Kemat Chemical Technology Co., Ltd. (Xi’an, China). Span-80 (AR) and anhydrous calcium chloride (CaCl_2_) (AR) were acquired from Beijing Innochem Technology Co., Ltd. (Beijing, China). Normal hexane (n-hexane, CAS 110-54-3) (GC, Purity ≥ 99%) was sourced from Shanghai Tengzhun Bio-Technology Co., Ltd. (Shanghai, China). PDMS (Purity ≥ 99%) was procured from Shanxi Jinyang Pharmaceutical Excipients Co., Ltd. (Shanxi, China). (3-Aminopropyl)triethoxysilane (APTES) (AR) and disodium hydrogen phosphate (AR) were purchased from Shanghai Macklin Biochemical Technology Co., Ltd. (Shanghai, China). Anhydrous ethanol (AR) was supplied by Tianjin Damao Chemical Reagent Factory (Tianjin, China). Peptone, beef extract powder, and agar powder were obtained from Beijing Aoboxing Bio-Tech Co., Ltd. (Beijing, China). Sodium chloride (AR) was acquired from Beijing Solarbio Science & Technology Co., Ltd. (Beijing, China). Potassium dihydrogen phosphate (AR) was purchased from Shanghai Aladdin Biochemical Technology Co., Ltd. (Shanghai, China). All experimental water was distilled water, and all reagents were of analytical reagent (AR) grade. *S. aureus* and *E. coli* were purchased from Beijing Beina Chuanglian Biotechnology Institute (Beijing, China). The CGF was supplied by Klarity Medical & Equipment Co., Ltd. (Guangzhou, China), featuring a 65/35 nylon/spandex warp-knitted structure with a fabric weight of 288 g/m^2^. The fabric specifications included: face side, 80 wales/50 mm (transverse) and 120 courses/50 mm (longitudinal); reverse side, 124 wales/50 mm (transverse) and 90 courses/50 mm (longitudinal).

### 2.2. Preparation of PDMS Microcapsules

**Figure 2:**
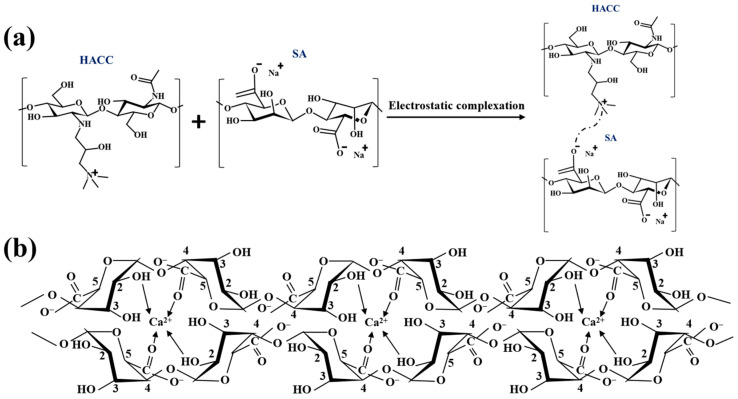
Schematic diagram of microcapsule wall material reaction. (**a**) Electrostatic interaction between HACC and SA. (**b**) “Egg box” model of calcium alginate gel.

This study employed the complex coacervation method to prepare microcapsules, the core principle of which involves the electrostatic attraction between positively charged quaternary ammonium groups in HACC and negatively charged carboxylate groups in SA. This interaction leads to phase separation and deposition on the surface of PDMS droplets, forming the preliminary wall structure of the microcapsules [28,30]. To further enhance the stability of the microcapsules, Ca^2+^ was introduced for cross-linking and solidification. This divalent cation specifically binds with the guluronic acid units in SA, forming a stable three-dimensional network structure through the “egg box” model. This cross-linking effect can significantly improve the compactness of the capsule wall and enhance its mechanical strength. The schematic diagram illustrating the electrostatic adsorption between HACC and SA, as well as the “egg box” structure formed by SA and CaCl_2_, is shown in Figure 2.

A quantity of 0.03 g HACC was dissolved in 300 mL of 1% (*v*/*v*) glacial acetic acid solution with continuous stirring in a 50 °C water bath until complete dissolution was achieved. Separately, 0.03 g SA was dissolved in 27 mL of distilled water under heating and stirring in a 50 °C water bath. To the SA solution, 0.06 g Tween-80 (functions as an effective emulsifier for the core material) was added and stirred until a homogeneous clear solution was obtained, followed by the addition of 0.06 g PDMS and 0.06 g Span-80 (as an emulsifier). This mixture was then homogenized at 15,000 rpm (1.5 W) for 5 min to form a stable, homogeneous emulsion. The HACC solution was maintained under magnetic stirring at 600 rpm while the emulsion was slowly introduced via syringe injection through a 6-gauge needle. Subsequently, a 5 wt% NaOH solution was added dropwise to adjust the pH to 6.0. The reaction mixture was continuously stirred at 25 °C for 30 min, after which 20 mL of 0.075 wt% CaCl_2_ solution was added dropwise. The resulting mixture was stirred in a constant-temperature water bath at 40 °C for 2 h to form the microcapsule suspension. Finally, the microcapsule suspension was washed, filtered, and freeze-dried for 12 h to obtain the PDMS microcapsule powder (PDMS-M).

### 2.3. Preparation of PDMS-M CGF

The PDMS-M finishing solution was prepared at a 50 g/L concentration in 100 mL volume. Four mL of APTES were mixed into this solution. The mixture was stirred continuously at 600 rpm speed for 10 min. Room temperature was maintained at 25 ± 2 °C during stirring. Pre-wetted CGF (10 cm × 20 cm) was used. These specimens were soaked in the prepared PDMS-M solution. Each specimen underwent three dip–roll operations. The finished CGF was then dried in an 80 °C oven. Then, the above dip–roll–bake operation was repeated. After the second cycle, the treated fabric was rinsed with distilled water. Final drying produced the finished PDMS-M CGF material. 

### 2.4. Preparation of PDMS-M-HACC CGF

A 30 g/L HACC solution was made using 100 mL water. The PDMS-M CGF was soaked in this solution and went through three dip–roll operations. The CGF was then dried at 80 °C. After drying, distilled water was used to rinse the fabric. Final drying produced PDMS-M-HACC CGF.

### 2.5. Drug Loading Capacity, Encapsulation Efficiency, and In Vitro Drug Release of Microcapsules

Since the PDMS was colorless and transparent, Sudan III was added as a colorant to prepare a 10 mg/mL stained PDMS-n-hexane solution for absorbance measurement. The maximum absorption peak was determined by full-wavelength scanning (200–800 nm) using a UV-visible spectrophotometer (Model 210S) (Analytik Jena AG, Jena, Germany) with n-hexane as the blank reference, followed by construction of a standard curve using corresponding concentration gradients. The dried PDMS-M was then immersed in n-hexane and subjected to sequential shaking, sonication, and centrifugation. The supernatant absorbance was measured to calculate the stained PDMS content. For release profiling, PDMS-M samples were incubated at 37 °C under open conditions, with sampling at 0 h, 12 h, 1 day, 1.5 days, 2 days, 3 days, 4 days, 5 days, 7 days, 10 days, 15 days, 20 days, 25 days, and 30 days time points. The drug loading capacity (DLC), encapsulation efficiency (EE), and relative cumulative release rate were calculated using the following equations:

$$\mathrm{DLC}\;(\%)\;=\;\frac{M_{1}}{M_{2}}\times 100\%$$

$$\mathrm{EE}\;(\%)=\frac{M_{1}}{M_{0}}\times 100\%$$

$$\mathrm{Relative}\mathrm{cumulative}\mathrm{release}\mathrm{rate}\;(\%)=(1-\frac{D_{1}}{D_{0}})\times 100\%$$

where *M*_1_, *M*_2_, and *M*_0_ represent the mass of the core material within microcapsules, the total mass of the microcapsules, and the initially fed core material mass, respectively, while *D*_1_ and *D*_0_ denote the drug loading capacity of microcapsules at a given time point and the initial drug loading capacity, respectively.

### 2.6. Antibacterial Property Test

The antibacterial CGF is a non-leaching antimicrobial textile. Antibacterial activity was tested using the GB/T 20944.3-2008 oscillation method [35]. *S. aureus* and *E. coli* served as test microorganisms. The antibacterial rate was calculated as:

$$\mathrm{The}\mathrm{antibacterial}\mathrm{rate}\;(\%)=\frac{A_{1}-A_{0}}{A_{1}}\times 100\%$$

where *A*_1_ and *A*_0_ represent the control sample’s bacterial count and the antibacterial or untreated fabric’s bacterial count.

### 2.7. Washing Fastness Test

Washing fastness was evaluated per FZ/T 73023-2006 “Antibacterial knitwear”—Appendix 3: Laundering Test Method for Antibacterial Fabric Samples [36]. This standard classifies antibacterial knitted fabrics into grades A, AA, and AAA based on washing resistance and antibacterial retention. PDMS-M CGF and PDMS-M-HACC CGF were tested against *S. aureus* and *E. coli* after 5, 10, 20, and 50 washes.

### 2.8. Wearability Test

A universal strength tester (Model 3369) (Instron Corporation, Norwood, MA, USA) measured tensile elastic recovery following FZ/T 70006-2022 “Stretch and recovery testing method for knitted fabrics [37].” The fabric specimen size was 200 mm × 50 mm, with a 100 mm × 50 mm effective testing area. Tests ran at 100 mm/min with three repeats in both the horizontal and vertical directions. The machine calculated average recovery rates.

The YG461H permeability tester (Ningbo Textile Instrument Factory, Ningbo, China) evaluated air flow per GB/T 5453-1997 “Textiles-Determination of the permeability of Fabrics to air [38].” The tests used a 20 cm^2^ specimen area under 100 Pa pressure. Multiple measurements yielded average permeability values.

The YG(B)216-II tester measured moisture transfer following GB/T 12704.2-2009 “Textiles-Test method for water-vapor transmission of fabrics—Part 2: Water method [39]” Circular 70 mm specimens were tested at 38 ± 2 °C and 50 ± 2% RH. Three test runs provided the final data.

The 101-2AB stiffness tester determined bending resistance as per GB/T 18318.1-2009 “Textiles-Determination of Bending Behavior—Part1: Incline method [40].” The size of the specimen was 20 mm × 250 mm, 6 specimens were prepared in horizontal and vertical directions, and the bending stiffness of the specimens was calculated.

### 2.9. Biocompatibility

#### 2.9.1. Cell Viability Experiment

As the PDMS-Ms serve as the core functional component of the finished CGFs and directly interact with cells, their potential biological effects were evaluated. The CGF material (nylon/spandex blended fabric) has been previously demonstrated to exhibit minimal cytotoxicity [41]. Since PDMS-Ms serve as the core functional component of the final CGF products and directly contact cells, this study evaluated their biocompatibility using CCK-8 assay and live/dead cell staining, with all procedures strictly complying with GB/T 16886.5-2017 national standard [42].PDMS-M samples were sterilized by UV irradiation for 12 h and incubated in serum-supplemented medium for 24 h to prepare extracts. NIH3T3 cells were seeded in 96-well plates and allowed to adhere for 24 h. The culture medium was then replaced with PDMS-M extracts for 72 h incubation. CCK-8 reagent was added, and absorbance was measured at 450 nm using a microplate reader (3000AL) (Shanghai SimpBio Biological Technology Co., Ltd., Shanghai, China). In this study, we referred to the method reported by Spano et al. [43] and used cisplatin (20 μm) treatment as the positive control group. Cell viability percentage was calculated as follows:

$$\mathrm{Cell}\mathrm{viability}\;(\%)=\frac{O_{1}-O_{0}}{O_{2}-O_{0}}\times 100\%$$

where *O*_1_, *O*_2_**,** and *O*_0_ represent the OD value of the experimental group, the OD value of the control group, and the OD value of the blank group. After completing the CCK-8 assay, cells were re-plated by seeding sterilized NIH3T3 cell suspension into 24-well plates. Following 24 h incubation for cell attachment, the culture medium was replaced with extraction medium, using 0.3% Triton-treated cells for 10 min as the positive control group. After 3 days of incubation, staining solution containing calcein-AM and PI was added and incubated at 37 °C for 15 min. After PBS washing, cell viability was observed and quantified under fluorescence microscopy. The cell survival rate was calculated as follows:

$$\mathrm{Cell}\mathrm{survival}\mathrm{rate}\;(\%)\;=\frac{L_{1}-L_{0}}{L_{1}}\times 100\%$$

where *L*_1_ and *L*_0_ represent the live cells and the dead cells.

#### 2.9.2. Skin Irritation Test—Direct Contact

Skin irritation potential was evaluated according to GB/T 16886.10-2017 “Biological evaluation of medical devices—Part 10: Tests for irritation and skin sensitization [44].” Six mice (6-week-old SD rats weighing 120–150 g) were used as experimental subjects, with each test material (1.5 cm×1.5 cm gauze impregnated with PDMS-M CGF or PDMS-M-HACC CGF extracts) applied to three mice. The samples were placed on shaved dorsal skin under occlusive dressing for 4 h. Skin reactions (erythema, edema, necrosis) were scored at 1 h, 24 h, 48 h, and 72 h post removal using standardized evaluation criteria.

### 2.10. Characterization Methods

The morphological characteristics of PDMS-M, PDMS-M CGF, and PDMS-M-HACC CGF were examined using a benchtop scanning electron microscope (Phenom XL) (Phenom-World B.V., Eindhoven, Netherlands) operating in secondary electron imaging mode. Elemental composition and distribution were analyzed with an Oxford energy-dispersive X-ray spectroscopy system (UltimMax 100) (Oxford Instruments Technology (Shanghai) Co., Ltd., Shanghai, China). Chemical group alterations in PDMS-Ms and functional group modifications on CGFs before and after treatment were investigated by Fourier transform infrared spectroscopy (Nicolet iS50) (Thermo Fisher Scientific Inc., Waltham, MA, USA). Molecular structure and atomic valence state information were characterized through X-ray photoelectron spectroscopy (AXIS SUPRA+) (Shimadzu Instruments (China) Co., Ltd., Shanghai, China) for both PDMS-M CGF and PDMS-M-HACC CGF samples.

## 3. Results and Discussion

### 3.1. Characterization of PDMS-Ms

**Figure 3:**
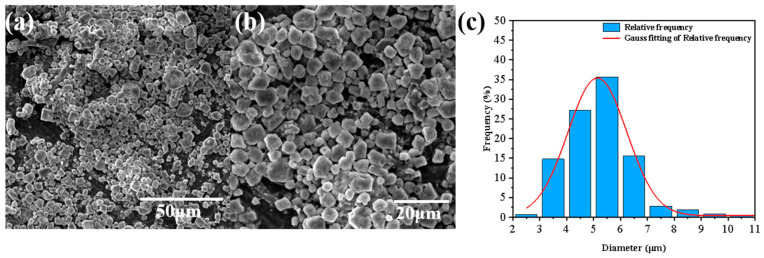
SEM images and particle size distribution diagrams of PDMS-Ms. (**a**,**b**) SEM images of PDMS-Ms. (**c**) particle size distribution diagrams of PDMS-Ms.

Figure 3a,b present SEM images of PDMS-Ms at 820× and 1900× magnification, respectively. The micrographs reveal that the majority of the microcapsules maintain well-defined irregular spherical morphology with intact structure, showing minimal interparticle agglomeration. As can be seen from the particle size distribution diagram in Figure 3c, the microcapsules predominantly exhibit particle sizes below 7 μm, with the majority of the microcapsules distributed in the range of 4–6 μm.

**Figure 4:**
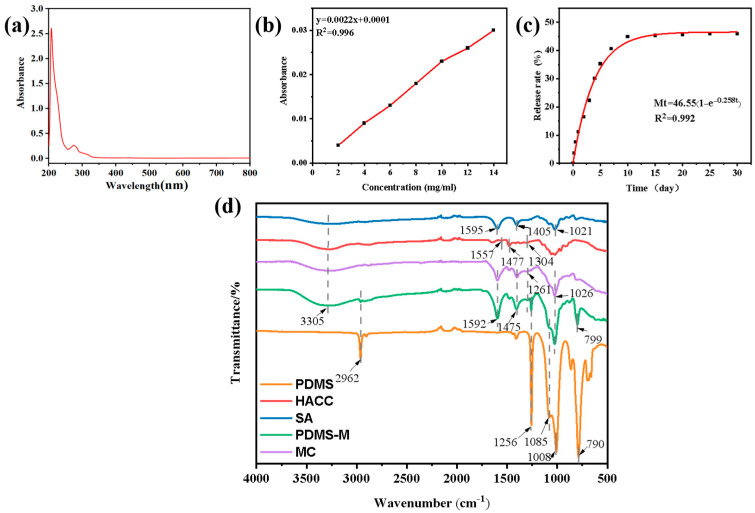
(**a**) Full-wavelength absorbance scanning of dyed PDMS-n-hexane solution. (**b**) Standard curve of stained PDMS-n-hexane solution at maximum absorption wavelength. (**c**) Time-dependent release profile of PDMS from PDMS-Ms. (**d**) FTIR spectra of PDMS-Ms, blank microcapsules (MCs), SA, HACC, and PDMS.

The maximum absorption peak at 503 nm is found by scanning the whole wavelength band of the stained PDMS-n-hexane solution (Figure 4a). The absorbance of the stained PDMS-n-hexane standard solution is measured at this maximum wavelength, with the n-hexane solution as the reference solution. The standard curve is plotted with absorbance as the ordinate and solution concentration as the abscissa (as shown in Figure 4b). After fitting, the standard curve equation of dyed PDMS is y = 0.0022x + 0.0001, where R^2^ = 0.996, which has a good linear fit. According to the standard equation, the absorbance can be obtained by an ultraviolet absorbance test, and then the corresponding concentration can be obtained.

The calculated encapsulation efficiency and drug loading capacity of the microcapsules reach 62.5% and 47.17%, respectively, meeting the general performance standards for microcapsules prepared by complex coacervation [28]. Figure 4c shows PDMS release kinetics from PDMS-Ms at 37 °C: initial rapid release (35% within 7 days) followed by sustained release (45% cumulative after 30 days). This biphasic profile results from the high initial concentration gradient driving rapid release, followed by the reduced concentration differential slowing subsequent release, achieving controlled long-term release. The release kinetics of the microcapsules is analyzed using the BoxLucas1 model, demonstrating first-order release characteristics (R^2^ = 0.992), with a maximum release of 46.55 ± 0.77%, a release rate constant of 0.259 ± 0.014 h^−1^, and a half-life of 2.68 h. Compared with the Higuchi model (R^2^ = 0.89), the BoxLucas1 model provides superior fitting performance, indicating that the system is not governed by simple diffusion control. SEM and FTIR analyses confirm that the release behavior is regulated by the dense structure of the wall material.

Figure 4d displays the FTIR spectra of PDMS-M components. Comparative analysis reveals a broad absorption peak at 3305 cm^−1^ in blank microcapsules (MCs), attributed to N-H and O-H stretching vibrations from HACC and SA. Characteristic peak shifts are observed: HACC’s C-H bending vibration (1477→1475 cm^−1^), -C-N^+^’s elastic vibration (1304→1261 cm^−1^), and SA’s -COO^−^ vibration (1595→1592 cm^−1^), confirming microcapsule formation through electrostatic interactions between HACC’s quaternary ammonium groups and SA’s carboxylate groups. In addition, by comparing the infrared spectra of the MCs and PDMS, the infrared spectra of PDMS-Ms show enhanced absorption peaks of 1085 cm^−1^, 1026 cm^−1^, and 799 cm^−1^ and a new characteristic absorption peak at 2962 cm^−1^ and 1256 cm^−1^, which reveals that PDMS is successfully encapsulated in the microcapsules. This is because the peaks at 1085 cm^−1^ and 1008 cm^−1^ are the stretching vibration of the silicon–oxygen bond Si-O of PDMS; the peaks at 2962 cm^−1^ and 1256 cm^−1^ are the -CH_3_ asymmetric stretching vibration and deformation vibration of PDMS, respectively; and the peak at 790 cm^−1^ is the Si-CH_3_ stretching vibration of PDMS. In summary, PDMS-Ms with SA and HACC as wall materials are successfully synthesized.

### 3.2. Characterization of PDMS-M CGF and PDMS-M-HACC CGF

**Figure 5:**
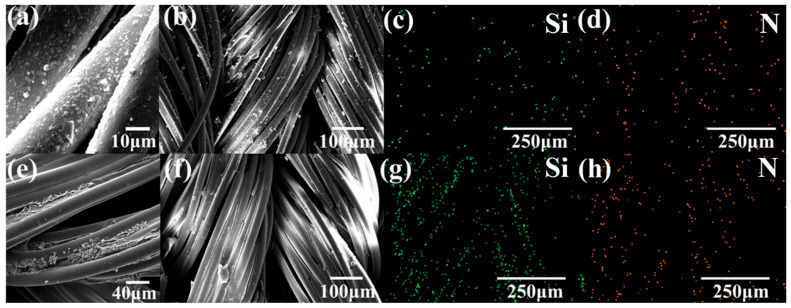
(**a**,**b**) SEM images of PDMS-M CGF. (**c**,**d**) EDS scan images of PDMS-M CGF. (**e**,**f**) SEM images of PDMS-M-HACC CGF. (**g**,**h**) EDS scan images of PDMS-M-HACC CGF.

Figure 5a,b present SEM images of PDMS-M CGF. Figure 5a demonstrates uniform distribution of microcapsules on fiber surfaces, confirming effective loading. Figure 5b reveals some irregularly shaped microcapsules attached between fibers, attributed to intense wall material reactions during preparation. Figure 5c,d display EDS scans of PDMS-M CGFs (without APTES addition to verify PDMS-M loading). Figure 5c shows distinct Si elemental mapping in fiber crevices and surfaces, confirming successful silicone microcapsule attachment. Figure 5d presents a uniform N element distribution, likely originating from chitosan quaternary ammonium salt in CGFs. Figure 5e,f exhibit SEM images of PDMS-M-HACC CGF. Figure 5f illustrates HACC forming a thin film encapsulating CGF fibers, while Figure 5e shows microcapsules and HACC aggregate in fiber gaps and surfaces, all enveloped by HACC film, demonstrating efficient co-loading. Figure 5g reveals a significantly increased Si signal compared to Figure 5c, due to APTES incorporation during the preparation of PDMS-M-HACC CGF. The N signal in Figure 5h is also significantly increased compared to the PDMS-M CGF Figure 5d. Combined with the surface in Figure 5e, the HACC coating on the surface of the CGF can clearly be seen. The above all indicate that HACC is loaded.

**Figure 6:**
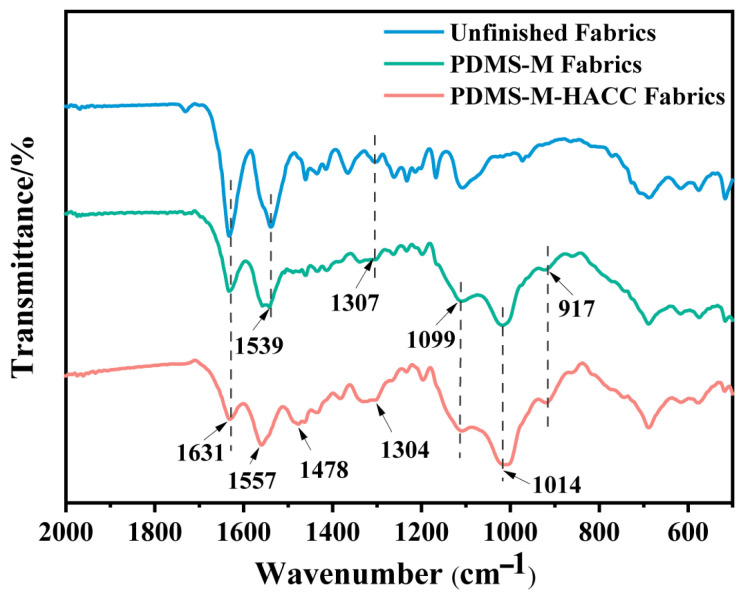
FTIR spectra of finished and unfinished CGF.

Figure 6 compares FTIR spectra of PDMS-M CGF, PDMS-M-HACC CGF, and untreated CGF. FTIR spectra of PDMS-M CGF show characteristic peaks at 1307 cm^−1^ (C-N stretch/N-H bend), 1539 cm^−1^ (C-N-H bend), and 1631 cm^−1^ (C=O stretch). APTES hydrolysis generates Si-O-Si (917 cm^−1^) and Si-O-C (1099/1014 cm^−1^) peaks through condensation reactions between silanol groups and hydroxyl groups on CGF/HACC/SA, confirming successful microcapsule–CGF conjugation. PDMS-M-HACC CGF exhibits enhanced characteristic peaks at 1478 cm^−1^ (C-H bend) and 1304 cm^−1^ (-C-N^+^ stretch), which both belong to the characteristic peaks of HACC, indicating that HACC is loaded onto the PDMS-M CGF and that the PDMS-M-HACC CGF is successfully prepared.

**Figure 7:**
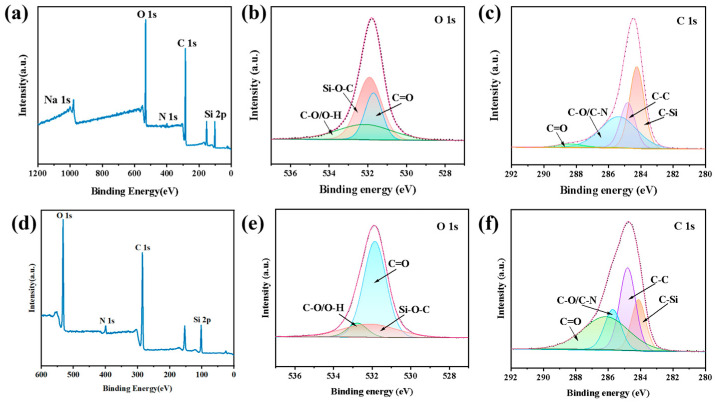
XPS spectra of PDMS-M CGF and PDMS-M-HACC CGF. (**a**–**c**) XPS spectra of PDMS-M CGF. (**d**–**f**) XPS spectra of PDMS-M-HACC CGF.

XPS survey scan Figure 7a confirms the presence of APTES, SA, HACC, and CGF components through Na 1s, O 1s, N 1s, C 1s, and Si 2p signals. In Figure 7b, the binding energies at 531.84 eV, 532.07 eV, and 532.76 eV correspond to the C=O, Si-O-C, and C-O/O-H bonds, respectively. The binding energies at 284.12 eV, 284.80 eV, 285.72 eV, and 286.13 eV correspond to the C-Si, C-C, C-O/C-N, and C=O bonds, respectively, in Figure 7c. The emergence of Si–O–C, C–N, and C–Si bonds in Figure 7b,c indicates that APTES undergoes a condensation reaction with the hydroxyl groups of the microcapsule wall material and the CGF to form chemical bonds, which fully proves that APTES successfully grafts the surface of microcapsule wall material and CGF. As shown in the XPS survey spectrum of Figure 7d, the presence of O 1s, N 1s, C 1s, and Si 2p signals confirms the existence of PDMS-M, HACC, and CGF in the PDMS-M-HACC CGF. In Figure 7e, the intensities of the C=O and -OH peaks, and in Figure 7f, the intensities of the C-O and C=O peaks, are all increased compared to the corresponding valence bond peaks in Figure 7b,c, indicating an increase in the hydroxyl and carbonyl contents of HACC. Further analysis of the semi-quantitative data of the O 1s and C 1s spectra shows that the proportion of quaternary ammonium salt peaks increases by 30% and the proportion of C=O peaks increase by 43%. This all proves the increase in the content of HACC in PDMS-M-HACC CGF, indicating that HACC is successfully loaded.

### 3.3. Antimicrobial Effect and Washing Fastness of PDMS-M CGF and PDMS-M-HACC CGF

**Figure 8:**
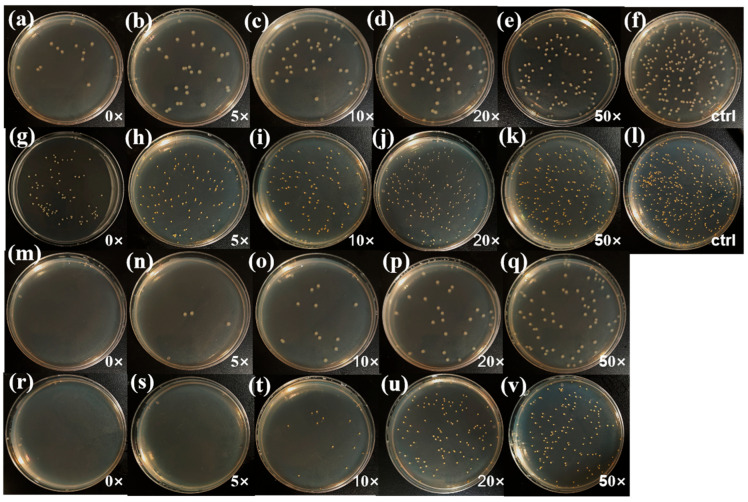
Antibacterial and washing fastness test results of PDMS-M CGF and PDMS-M-HACC CGF. (**a**–**f**) Antibacterial activity of PDMS-M CGF against *E. coli* (**a**–**e**) after 0–50 washes, (**f**) blank control. (**g**–**l**) Antibacterial activity of PDMS-M CGF against *S. aureus* (**g**–**k**) after 0–50 washes, (**l**) blank control. (**m**–**q**) Antibacterial activity of PDMS-M-HACC CGF against *E. coli* (**m**–**q**) after 0–50 washes. (**r**–**v**) Antibacterial activity of PDMS-M-HACC CGF against *S. aureus* (**r**–**v**) after 5–50 washes.

**Figure 9:**
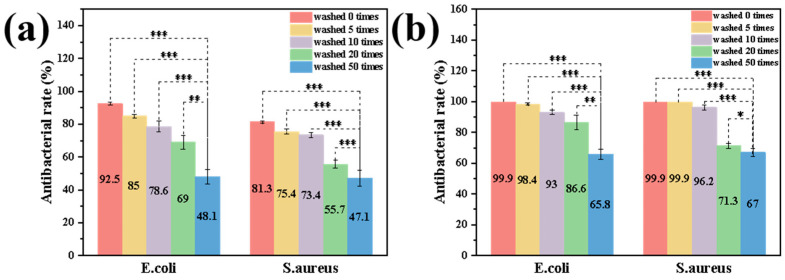
Antibacterial and washing fastness test results of PDMS-M CGF and PDMS-M-HACC CGF against *E. coli*. and *S. aureus*. (**a**) Antibacterial and washing fastness test results of PDMS-M CGF. (**b**) Antibacterial and washing fastness test results of PDMS-M-HACC CGF. (*: *p* ≤ 0.05, **: *p* ≤ 0.01, ***: *p* ≤ 0.001, *p* > 0.05: the result is not statistically significant.)

As shown in Figure 8 and Figure 9, both PDMS-M CGF and PDMS-M-HACC CGF maintain statistically significant antibacterial activity after 0, 5, 20, and 50 washing cycles compared to the blank control (*p* < 0.001), though their efficacy is notably diminished after 50 cycles (*p* > 0.001). Figure 8a–f presents the antimicrobial test results of PDMS-M CGF against *E. coli* after various washing cycles, while Figure 8g–l shows corresponding results against *S. aureus*. Bacterial counts from Figure 8 are calculated to determine the antibacterial rates of PDMS-M CGF against both *S. aureus* and *E. coli* before and after washing, as summarized in Figure 9a. The unwashed PDMS-M CGF demonstrates antibacterial rates of 92.5% against *E. coli* and 81.3% against *S. aureus* Figure 9a. After five washing cycles, these values decrease to 85% (*E. coli*) and 75.4% (*S. aureus*), indicating partial removal of surface-adsorbed or weakly-bound PDMS-Ms. Overall, the antibacterial performance of PDMS-M CGF fails to meet the grade A standard for either bacterial strain, suggesting the need for further improvement. Figure 8m–q,r–v display the antimicrobial performance of PDMS-M-HACC CGF against *E. coli* and *S. aureus*, respectively, after different washing cycles. Corresponding antibacterial rates are shown in Figure 9b. As can be seen from Figure 9b, the antibacterial rate of unwashed PDMS-M-HACC CGF against both *E. coli* and *S. aureus* is 99.9%. Overall, after different washing times, the PDMS-M-HACC CGF has significantly higher antibacterial rates against both *E. coli* and *S. aureus* than the PDMS-M CGF, and the washing fastness is strengthened. Following 20 washing cycles, the PDMS-M-HACC CGF maintains an antibacterial rate of 86.6% against *E. coli* (exceeding grade AA ≥ 70%). The antibacterial rate of PDMS-M-HACC CGF washed 50 times against *E. coli* is 65.8%, which is close to grade AAA (≥70%) of antibacterial knitted fabrics. Figure 9b shows that the antibacterial rate of PDMS-M-HACC CGF against *S. aureus* after 10 washes is 96.2%, close to grade A of antibacterial knitted fabrics (≥99%); the antibacterial rate against *S. aureus* after 20 washes is 71.3%, close to grade AA of antibacterial knitted fabrics (≥80%).

### 3.4. Wearing Performance of PDMS-M CGF and PDMS-M-HACC CGF

**Figure 10:**
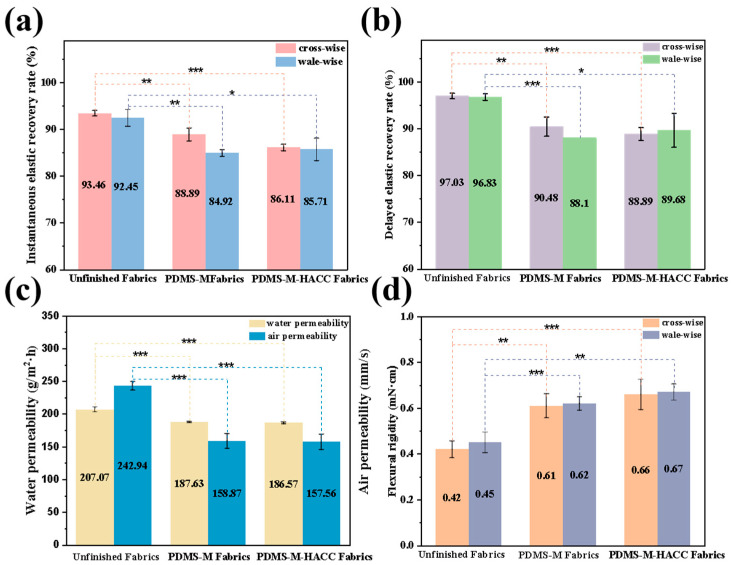
Wear resistance test results of unfinished fabric, PDMS-M CGF, and PDMS-M-HACC CGF. (**a**) Instantaneous elastic recovery rate. (**b**) Delayed elastic recovery rate. (**c**) Moisture permeability and air permeability. (**d**) Flexural rigidity. (*: *p* ≤ 0.05, **: *p* ≤ 0.01, ***: *p* ≤ 0.001, *p* > 0.05: the result is not statistically significant.)

The wearing performance of finished CGFs is evaluated through tensile elastic recovery, air permeability, moisture permeability, and bending stiffness tests (Figure 10). Figure 10a,b demonstrate decreased elastic recovery in both PDMS-Ms and PDMS-M-HACC CGFs compared to untreated fabric, attributed to microcapsule deposition in fiber interstices and on fiber surfaces increasing inter-fiber friction during stress recovery. PDMS-M CGF shows marginally higher delayed/instantaneous elastic recovery rates than PDMS-M-HACC CGF, likely due to additional HACC-induced friction. Notably, all delayed elastic recovery rates exceed 88%, indicating acceptable minimal impact from antimicrobial finishing.

Figure 10c reveals progressively reduced air permeability with PDMS-M and HACC finishing, resulting from microcapsule penetration into fabric pores decreasing porosity. While moisture permeability shows a slight reduction compared to untreated fabric, the magnitude remains insignificant. Figure 10d displays substantially increased bending stiffness for PDMS-M CGF compared to untreated fabric, which is further enhanced in PDMS-M-HACC CGF. This stiffening effect originates from microcapsule distribution throughout the fabric structure and their strong bonding to fibers, with HACC addition amplifying inter-fiber friction.

### 3.5. Biocompatibility of PDMS-M CGFs and PDMS-M-HACC CGFs

#### 3.5.1. Cell Viability Experiment Results and Discussion

**Figure 11:**
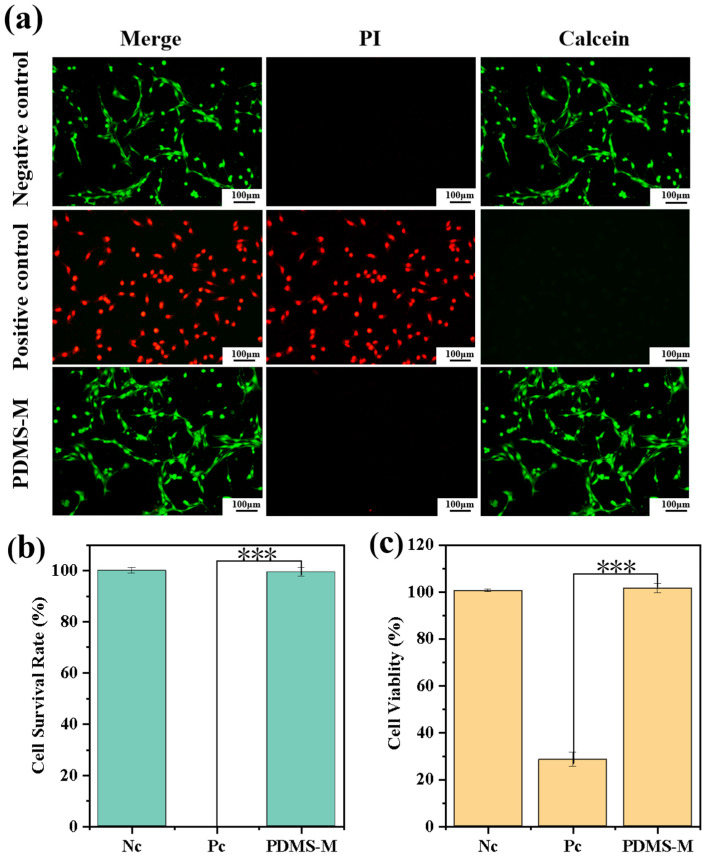
Cell viability assay. (**a**) Live/dead staining of NIH3T3 cells co-cultured with PDMS-Ms (green: viable cells; red: dead cells). (**b**) Quantitative cell survival rate from live/dead assay. (**c**) Cell viability measured by CCK-8 assay after PDMS-M exposure. (Nc: negative control, Pc: positive control). (***: *p* ≤ 0.001, *p* > 0.05: the result is not statistically significant.)

Live/dead staining reveals comparable cell distribution and viability between the PDMS-M group and negative control groups, with the positive control group exhibiting extensive red fluorescence staining of dead cells (Figure 11a). The PDMS-M group shows no significant difference compared to the negative control group (*p* > 0.05) but exhibits statistically significant differences versus the positive control group (*p* < 0.001). This confirms that the PDMS-M extract maintains membrane integrity without increasing cell death. The CCK-8 assay demonstrates equivalent metabolic activity between the PDMS-M group and negative control groups (Figure 11c), with no statistically significant difference (*p* > 0.05), while showing significant differences compared to the positive control group (*p* < 0.001). This indicates unimpaired dehydrogenase activity and normal cell proliferation. The statistically significant difference in the positive control group validates the reliability of the detection system. These complementary assays—assessing metabolic function (CCK-8) and morphological integrity (live/dead staining)—collectively establish PDMS-Ms’ excellent biocompatibility with NIH3T3 cells, supporting potential biomedical applications.

#### 3.5.2. Skin Irritation Test Results and Discussion

**Figure 12:**
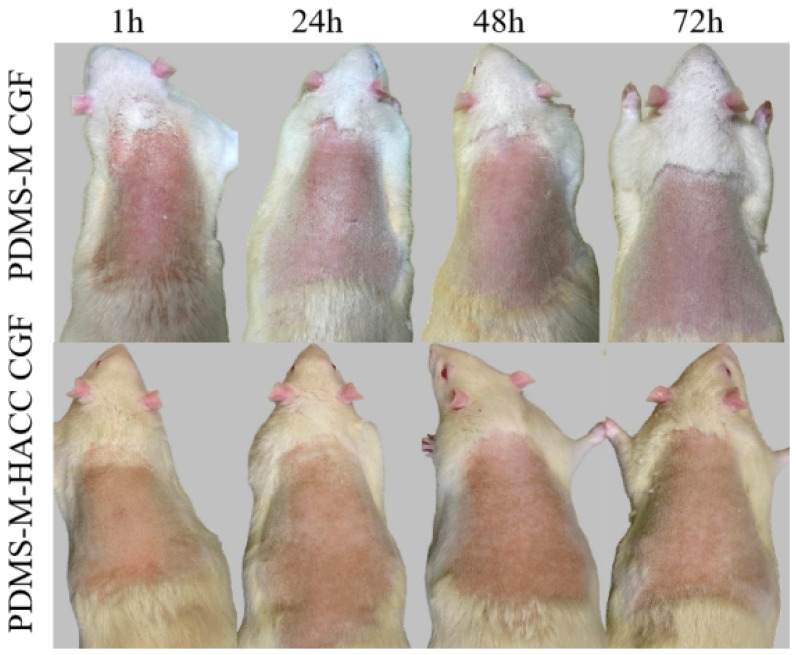
Skin tissue response in mice after patch application.

Comprehensive observation of mouse skin at (1 ± 0.1) h, (24 ± 2) h, (48 ± 2) h, and (72 ± 2) h post removal reveals no erythema, edema, necrosis, or other adverse reactions (Figure 12). The experimental groups show no exceeding reactions compared to controls, with a skin irritation score of 0. These results conclusively demonstrate the non-irritating nature and excellent biocompatibility of both PDMS-M CGF and PDMS-M-HACC CGF under test conditions.

## 4. Conclusions

In order to prepare functional CGF with dual scar-suppressing and antimicrobial properties, this study designed and prepared microcapsules and functional-finished CGF, and investigated the properties of the microcapsules and the finished CGF. The following are the main research conclusions:

(1) The microcapsules exhibit spherical morphology with uniform size distribution and excellent surface integrity. FTIR spectroscopy and UV-Vis analysis confirm successful PDMS-M synthesis, while release studies demonstrate biphasic release kinetics—initial rapid release followed by sustained release, retaining 45% of the core material after 30 days.

(2) SEM and EDS analyses verify successful PDMS-M CGF fabrication. FTIR and XPS characterization results confirm APTES-mediated covalent bonding between microcapsules and CGF. PDMS-M CGF shows 94.2% and 83.1% bacteriostatic rates against *E. coli* and *S. aureus*, respectively. SEM, FTIR, EDS, and XPS characterization results show that the content of HACC is significantly increased, indicating that HACC is successfully loaded and that PDMS-M-HACC CGF is successfully prepared. The antibacterial rates of PDMS-M-HACC CGF against *E. coli* and *S. aureus* are both 99.9%. PDMS-M-HACC CGF maintains an 86.6% antibacterial rate against *E. coli* after 20 washes (exceeding grade AA ≥70%) and 96.2% antibacterial rate against *S. aureus* after 10 washes (approaching grade A ≥99%).

(3) Compared to untreated fabric, PDMS-M CGF shows decreased tensile elastic recovery, air permeability, and softness, with further reduction in PDMS-M-HACC CGF, though all values remain within acceptable ranges. The moisture permeabilities of PDMS-M CGF and PDMS-M-HACC CGF decrease less than that of untreated fabric.

(4) CCK-8 assays and live/dead staining demonstrate excellent biocompatibility, with no significant cytotoxicity (*p* > 0.05). Skin irritation tests yield a score of 0, confirming the non-irritating properties and validating the materials’ safety for dermal applications.

The deficiency of this study lies in the inhibitory effect of finished CGF on scar hyperplasia relying on the literature rather than direct quantification. In terms of wear performance, the antibacterial finishing process has a certain impact on the elastic recovery, air permeability, and bending stiffness of the CGF. Although it is within an acceptable range, there is still room for optimization. Future work should focus on material optimization through novel formulations and processing technologies to further enhance patient comfort and therapeutic outcomes.

## Data Availability

The original contributions presented in the study are included in the article, further inquiries can be directed to the corresponding author.
